# Chemotactic motion of coronin-null cells with impaired actin turnover

**DOI:** 10.1186/s12860-026-00585-9

**Published:** 2026-03-26

**Authors:** Mary Ecke, Jana Prassler, Annette Müller-Taubenberger, Günther Gerisch

**Affiliations:** 1 Max Planck Institute of Biochemistry, Am Klopferspitz 18, D-82152 Martinsried, Germany; 2 Division of Cell Biology, Biomedical Center, LMU Munich, Großhaderner Str. 9, D- 82152 Planegg-Martinsried, Germany

**Keywords:** Actin dynamics, Blebbing, Blebbistatin, Chemotaxis, Coronin, *Dictyostelium discoideum*

## Abstract

**Background:**

Previous studies have shown that *Dictyostelium discoideum* cells lacking the actin-regulating protein coronin A have a large hyaline zone at the front of the cell. However, the coronin mutant cells can efficiently navigate in a gradient of chemoattractant by extending rounded protrusions from the hyaline zone. This study examines whether this zone is occupied by actin filaments, as was previously assumed, or if it is free of filamentous actin, as would be the case for typical blebs.

**Results:**

The lack of coronin A results in a large hyaline region in the anterior part of mutant cells, from which the endoplasmic reticulum is displaced. This zone is populated with filamentous actin. Despite this broadened front, the coronin-null cells can respond in a gradient of chemoattractant even when they lack actin-based structures such as filopodia or lamellipodia. During re-orientation into a changed direction of the gradient, the mutant cells form rounded protrusions in various directions, of which the one pointing to the direction of the gradient will expand and become the new front. Contact with a substratum is not necessary for a protrusion to be formed in the right direction. These results illustrate the altered but efficient chemotactic responses of cells under conditions of diminished actin filament turnover.

**Conclusions:**

Despite an impaired actin filament turnover, *Dictyostelium discoideum* cells lacking coronin A respond to a gradient of chemoattractant by extending rounded protrusions from their large hyaline fronts. These protrusions are rich in filamentous actin, and filopodia or lamellipodia are not required. This mode of chemotactic migration is different from a bleb-driven mode described for *Dictyostelium* and other eukaryotic cells.

**Supplementary Information:**

The online version contains supplementary material available at 10.1186/s12860-026-00585-9.

## Background

In eukaryotic cells, migration in a gradient of chemoattractant is usually accomplished by formation of lamellipodia or filopodia, protrusions formed by the actin cytoskeleton that are directed toward the source of attractant. Re-orientation in changing gradients may be achieved by turning of the present front or by induction of a new front at the rear or any site of the cell [1–3]. These chemotactic responses are mediated by signal transduction mechanisms [4–8] that guide the filamentous actin structures toward the direction of the gradient of attractant.

The network of filamentous actin is controlled by a balance of actin polymerization and depolymerization. Polymerization factors, the Arp2/3 complex [9–11] and formins [12, 13], compete with depolymerizing factors, keeping the actin layer of the cell cortex at limited thickness. The best-studied actin depolymerizing system is the ADF (Actin Depolymerizing Factor) – cofilin system [14–17]. Coronin, a WD40 repeat protein [18] with a N-terminal seven-bladed β-propeller structure [19, 20], has been proposed to be involved in cofilin-mediated actin depolymerization [11]. Cofilin has been shown to cause a supertwist along the actin filament toward the minus end [21, 22], and coronin to destabilize the actin filaments by binding preferentially to the super-twisted region [23].

Coronin was initially described in *Dictyostelium discoideum*, and subsequently coronin proteins were found in many eukaryotic species from yeast to humans. The human coronin family contains seven isoforms. Here we refer to *Dictyostelium* coronin A (CorA), rather than coronin 7 which has been discovered later [24]. In the first report on coronin, aberrations in cytokinesis of coronin-null mutants suggested a role for coronin in the regulation of actin polymerization [25]. An increased content of F-actin in coronin-null cells supported an impairment of actin depolymerization [26]. These mutant cells have a broad hyaline zone in the front region and move by the protrusion of this zone [27]. The shape of chemotaxing coronin-null cells with their broad hyaline front region, is also compatible with a deficiency in actin depolymerization [18, 28].

In this study, we have investigated the responses of coronin-null cells stimulated by chemoattractant. At first glance, coronin-null cells appear to behave similar as *Dictyostelium* wild-type cells do under certain conditions [29–31]. Blebbing is a saltatory protrusion of the cell membrane caused by local separation of the membrane from the underlying actin cortex [32–35]. The force for blebbing in *Dictyostelium* cells is generated by myosin II-dependent contractility: myosin II-null cells do not bleb [36, 37], and neither do wild-type cells treated with the myosin II-ATPase blocker blebbistatin [38]. The fluorescence intensity of an actin reporter is less at the membrane of a bleb than in the remaining cortical layer [39] Significant amounts of F-actin only return to the membrane once the expansion of a vesicle has ceased. Together with the requirement of myosin II, these data suggest that blebs form due to an increase in hydrostatic pressure that extends the cell in regions where the cortex is weakened. Blebbing of *Dictyostelium* cells in response to a sudden increase in chemoattractant has been studied by Langridge and Kay [39], and Yoshida and Soldati [36]. They emphasized that blebbing motility in *Dictyostelium* must be considered an alternative to filopodia and lamellipodia formation, not only under special conditions [31]. These studies revealed that there are no detectable changes in cell volume or surface area during blebbing. They also established that blebbing motility is consistent with efficient chemotactic responsiveness. Different from the blebs are spherical cell-surface protrusions formed in cells that constitutively overexpress active RacB [40]. The lumen of these protrusions is rich in filamentous actin, and their formation is independent of myosin II.

## Methods

### Cell strains and culture conditions

*Dictyostelium discoideum* cells of the strain AX2-214 or coronin A-null mutant cells (HG1569) derived from this parent strain, which expressed green and red fluorescent proteins (GFP and RFP, respectively) were used for all experiments (see Table 1). Cells were cultivated in nutrient medium as described [41], supplemented with 10 µg/ml blasticidin S (B10) (Gibco, Life Technologies Corporation, Grand Island, NY, USA), 10 µg/ml geneticin (G10) (Sigma-Aldrich, St. Louis, MO, USA), and/or 33 µg/ml of hygromycin B (H33) (Calbiochem, Merck, Darmstadt, Germany) in plastic Petri dishes at 21 ± 2 °C as indicated in Table 1.

**Table 1:** Strains and references for GFP- and RFP-labeling

Strain	GFP Label	RFP Label	Resistance*	References
AX2-214	CoroninA-GFP	mRFPM-LimEΔ	G10 / B10	[42, 43]
AX2-214	Calnexin-GFP	mRFPM-LimEΔ	G10 / B10	[42, 44]
AX2-214	GFP-LimEΔ	--	B10	[45]
CoroninA-null HG1569	GFP-Actin	mRFPM-LimEΔ	B10 / H33 Knockout G10	[42, 46]
CoroninA-null HG1569	GFP-LimEΔ	Calnexin-mCherry	B10 / H33 Knockout G10	[45, 47]
CoroninA-null HG1569	GFP-LimEΔ	--	B10 Knockout G10	[45]

*B10 = 10 µg/ml blasticidin S / H33 = 33 µg/ml hygromycin B / G10 = 10 µg/ml geneticin

### Sample preparation for chemotactic stimulation with a micropipette filled with cAMP and confocal image acquisition

AX2 wild-type or coronin-null cells with respective RFP- and GFP-label were rinsed off the Petri dishes with 17 mM Na/K-phosphate buffer (PB), pH 6.0, after the nutrient medium was removed. Following two washes with ice-cold PB, with a centrifugation step in between 200 g at 4°C, cells were adjusted to 1 × 10^7^ cells per ml in PB, transferred to a small Erlenmeyer flask, and starved under shaking conditions at 160 rpm at 21 ± 2°C. After 1 hour, cells were pulsed with a final concentration of 30 nM cAMP (3’,5’-cyclic adenosine monophosphate). After 5 to 6 h these cells were gently resuspended. 20 µl of the cell suspension was transferred to a HCl-cleaned cover-glass bottom dish (FluoroDish, WPI INC., FL, USA) supplemented with 3 ml PB, and cells were allowed to settle down for about 15 min.

A micromanipulator (Eppendorf SE, Hamburg, Germany) was mounted on the microscope as described below. An Eppendorf Femtotip micropipette was filled with 0.1 mM cAMP in PB and connected to the manipulator. The tip was brought to the focus plane of the cells. A detailed description of this procedure can be found in a book chapter by Ecke and Gerisch [48].

Live cell imaging was done with an inverted Zeiss LSM 780 confocal microscope equipped with a Plan-Apochromat 40x/NA 1.4 water immersion objective (Zeiss AG, Oberkochen, Germany) at 23^o^ C. If not indicated otherwise, six confocal z-planes were obtained from the substrate-attached surface to the top of the cell at maximum speed.

### Fixation during chemotactic stimulation followed by fluorescent labelling with phalloidin

Coronin-null cells labelled with GFP-LimEΔ were prepared as described above, transferred to a HCl-cleaned gridded glass bottom dish (Grid-50 glass bottom dish, ibidi GmbH, Gräfelfing, Germany) supplemented with 3 ml PB, and left to settle down at 21 ± 2 °C. The gridded glass bottom dishes were used to locate reacting cells after the fixation and mounting process. Cells were stimulated with a cAMP-filled micropipette. After the cells responded to the pipette, 3 ml of a double concentrated paraformaldehyde-picric acid fixative was added to the dish [49]. After 15 min, the fixative was removed and the fixed cells were washed with 10 mM PIPES, incubated in PBS with 100 mM glycine followed by 70% ethanol for 10 min. This was followed by several washing steps with PBS containing 0.1% fish gelatin and 0.5% BSA (PBG). Alexa fluor™ 568-phalloidin (Invitrogen by Thermo Fisher Scientific, Massachusetts, USA) was used to stain filamentous actin according to the manufacturer’s protocol with a dilution of 1/400 in PBG from a 66 µM DMSO stock solution. After 2 h, the preparation was washed with PBS and mounted in Gelvatol supplemented with 1,4-diazabicyclo[2.2.2]octane (DABCO) as an antioxidant and quencher of singlet oxygen that improves the lifetime of the fluorophores. The fixed cells labelled with Alexa fluor 568™-phalloidin were imaged with a Plan-Apochromat 64x/NA 1.46 oil immersion objective (Zeiss AG, Oberkochen, Germany) on a Zeiss LSM 780 confocal microscope, and z-planes were recorded at 0.1 μm intervals from the glass bottom to the top of the cells.

### Confocal image acquisition and confocal reflection interference contrast microscopy (RICM)

To monitor the shape of the cell membrane in interaction with the glass surface during chemotaxis of coronin-null cells labeled with GFP-LimEΔ and calnexin-mCherry (CNX-mCherry), RICM was applied using a Zeiss LSM 780 confocal microscope equipped with a Plan-Apochromat 40x/NA1.4 DIC M27 oil immersion objective (Zeiss AG, Oberkochen, Germany). Reflection images were generated with a far red 633-nm HeNe-laser. An MBS T80/R20 dichroic mirror, a 586–674 nm emission filter and a GaAsP photomultiplier for detection of the reflected light were used [50–52]. Parallel to RICM images, bright-field images were acquired. Sequentially to RICM, the fluorescence channels for GFP (excitation 488 nm, emission 500–560 nm) and mCherry (excitation 561 nm, emission 570–640 nm) were recorded.

### Chemoattractant stimulation of blebbing, suppression of blebbing and stimulation with a micropipette in the presence of (S)-3’-hydroxy-blebbistatin, a myosin II-inhibitor

(S)-3’-hydroxy-blebbistatin (Cayman Chemical, Michigan, USA) was used to inhibit myosin II- ATPase activity and thereby blebbing was suppressed. (S)-3’-hydroxy-blebbistatin is a 30-fold higher water soluble, non-phototoxic, non-fluorescent blebbistatin derivative for cell-based assays [53, 54]. To test the concentration of (S)-3’-hydroxy-blebbistatin required for suppression of blebbing in *Dictyostelium* AX2 wild-type, cells labeled with GFP-LimEΔ were starved and pulsed for 5 to 6 hours, diluted to 1 × 10^6^ cells per ml and transferred to a HCl-cleaned 8-well glass bottom dish (ibidi GmbH, Gräfelfing, Germany). After cells settled down, imaging started and blebbing was induced with 4 µM cAMP final. Before blebs appear in a 30 to 50 s range after addition of cAMP, a change of F-actin distribution was observed like described previously by Langridge and Kay [39]. In the next well with cells, PB was replaced by 200 µM (S)-3’-hydroxy-blebbistatin in PB, diluted from a 100 mM stock in DMSO, and incubated for 1 h. After this pre-treatment, application of 4 µM cAMP did not cause blebbing of AX2 wild-type cells (Figure S2). The same experiment was conducted with coronin-null cells.

After testing these conditions, the starved and pulsed coronin-null cells were transferred to a glass-bottom dish. They were left for 15 min to settle down and then incubated for 1 h in 200 µM (S)-3’-hydroxy-blebbistatin. Then, coronin-null cells were chemotactically stimulated with a micropipette filled with cAMP as described above and recorded.

### Data processing

To process the images, the image-processing package Fiji (http://Fiji.sc/Fiji) on the basis of ImageJ (http://imagej.nih.gov/ij) was used [55]. If not indicated otherwise, average projections of series of confocal planes are shown. At long periods of imaging, the RFP-label tended to bleach, and we corrected the signal in the red channel with the “Bleach Correction” plugin in “Simple Ratio Mode”.

To evaluate the hyaline zone of AX2 wild-type and coronin-null mutants with and without treatment with the myosin II-inhibitor (S)-3’-hydroxy-blebbistatin, cells labelled with either GFP-CNX or CNX-mCherry together with GFP- or RFP-LimEΔ were used. The summed z-planes of the LimEΔ-label were used for the evaluation of the total areas of the cells, and the areas occupied by the CNX-label were used for the non-hyaline part of the cells. They were encircled with the “Polygon selection” tool and areas were evaluated with the “Measure” tool command in µm^2^. Data was transferred to Excel for calculation of the percentage of area of the hyaline zone in respect to the total cell area. 10 cells of wild-type, 10 cells for coronin-null without blebbistatin, and 10 cells for coronin-null with blebbistatin were measured (Fig. 5A).

To measure velocity of directional movement of coronin-null mutant cells stimulated with cAMP through a micropipette with and without (S)-3’-hydroxy-blebbistatin, the front of 15 mutant cells without and 8 mutant cells treated with (S)-3’-hydroxy-blebbistatin were tracked using the “Manual tracking” tool. The data was transferred to Excel and average velocity was calculated (Table 2).

The data of fixed, phalloidin-stained cells was deconvolved using the program Hyugens Essential (https://svi.nl/Huygens-Essential). The “Orthogonal View” tool was used to show the 3D distribution of Alexa Fluor™ 568 phalloidin in fixed cells in xz- and yz-direction. Grey values of the images in Fig. 3B are presented in a fire Look Up Table (LUT).

**Fig. 1:**
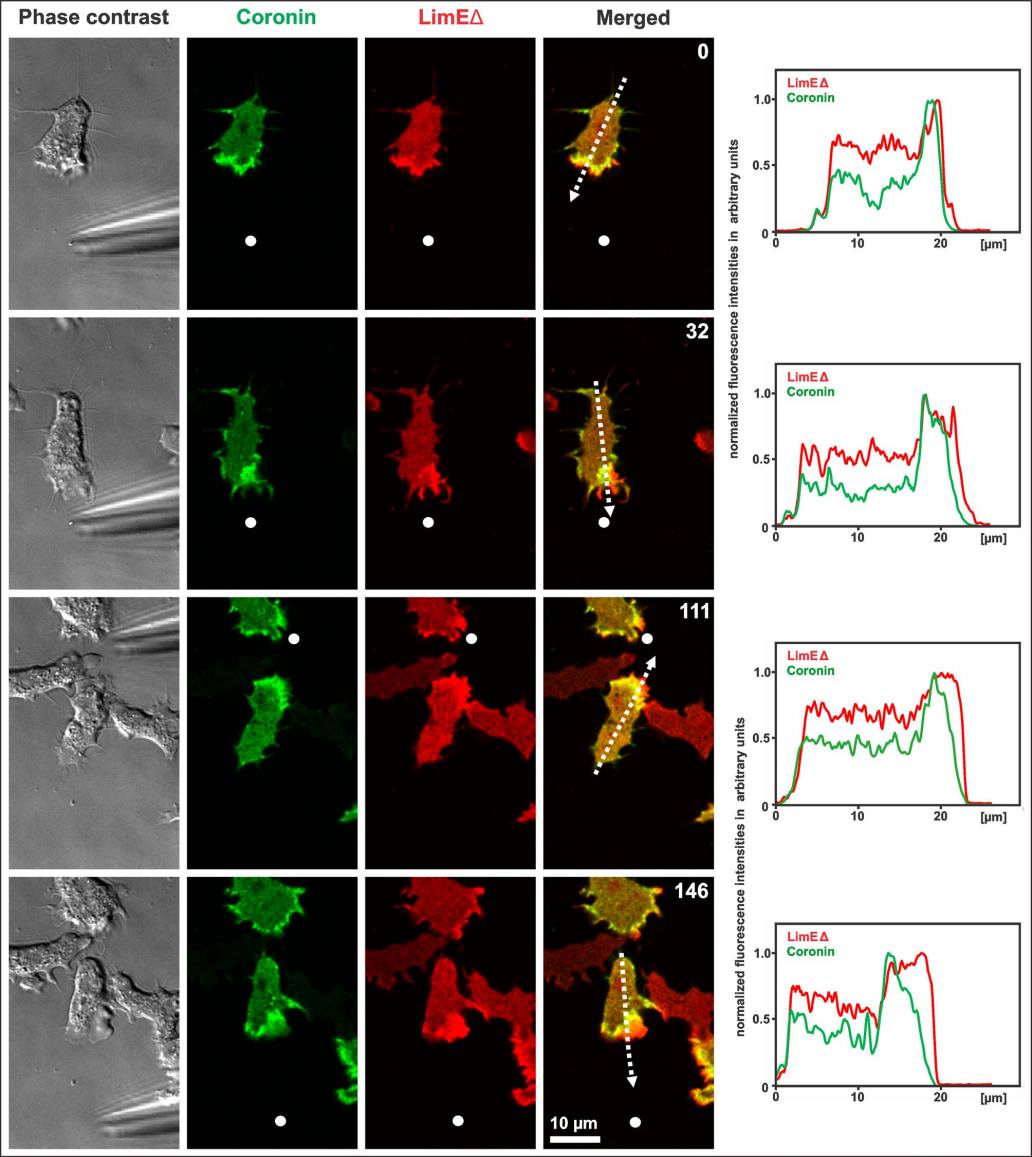
Chemotaxis of aggregation-competent AX2 wild-type cells expressing coronin-GFP (green) and mRFP-LimEΔ (red). The cells react to a gradient of cAMP that is built around the tip of the micropipette (white dot). After repositioning of the pipette, the cells reorient likewise. Single planes at the substrate-attached bottom are shown. Corresponding line scans of the fluorescence intensities of coronin (green) and LimEΔ (red) from the tail to the front of the cells are presented on the right side of the merged images indicated with a white, dotted arrows. LimEΔ is slightly accumulating at the cell front before coronin. Time is indicated in seconds. Bar, 10 μm. This figure corresponds to the movie shown in additional Movie S1

**Fig. 2:**
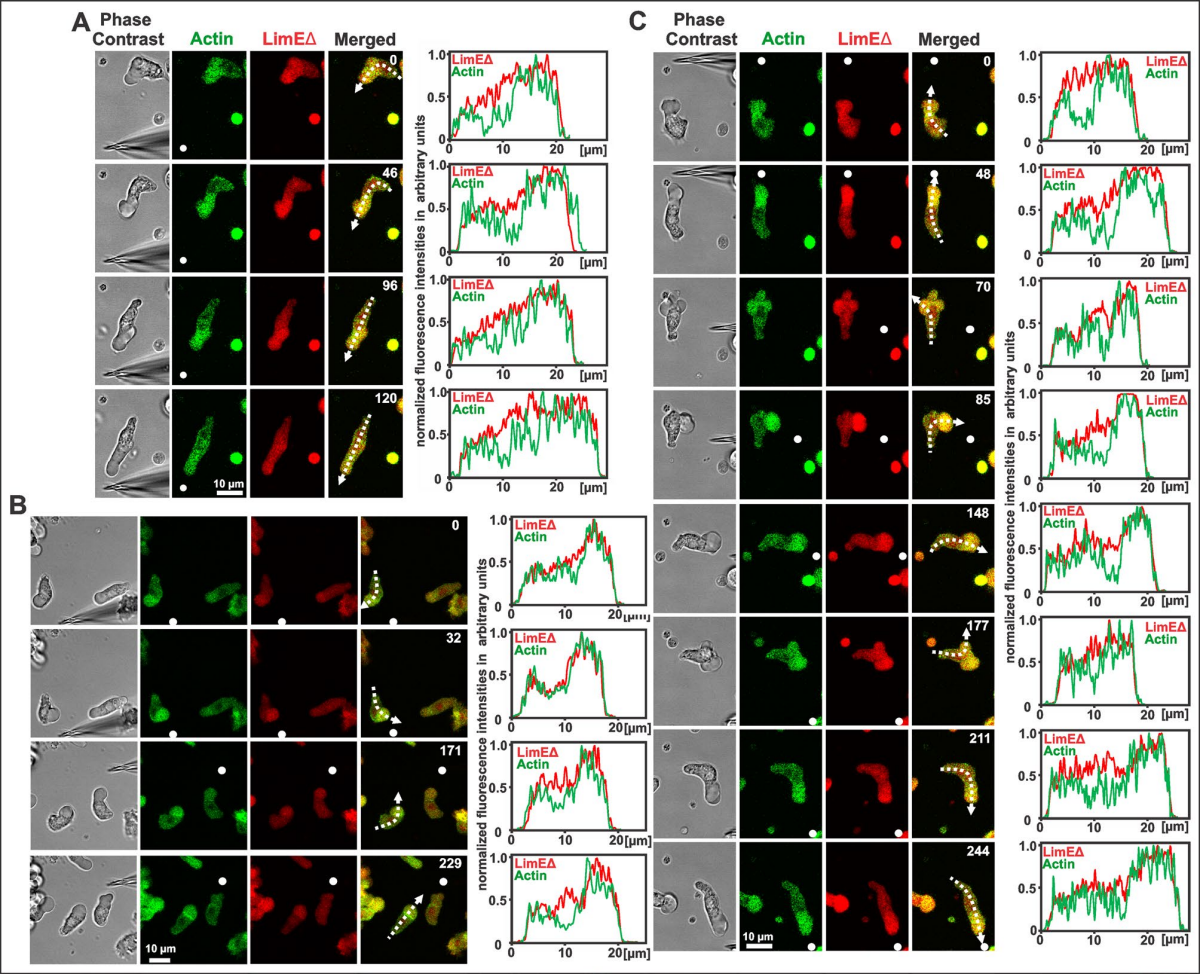
Three sequences of chemotaxing coronin-null cells that expressed actin-GFP (green) and LimEΔ (red). The cells responded to a gradient of cAMP released from the tip of a micropipette (white dots in the fluorescence images). On the right side of the images corresponding line scans of the fluorescence intensities, indicated by dotted, white arrows, are presented in the merged images. (**A**) One cell responding to the chemoattractant gradient with a large hyaline zone that is clearly labeled with actin-GFP and LimEΔ. (**B**) Two cells respond with a round front turning the anterior hyaline zone into the direction of the gradient. (**C**) Upon changing the position of the micropipette, the tail continued to move in the previous direction of the gradient, while the front region initially did not only make protrusions into the direction of the new gradient (70 s). However, only the protrusions in the correct direction persisted to become the new front region (85 s). The line scans of the fluorescence intensities of the non-persisting protrusions in the 70-s frame and 177-s frame of (**C**) show that actin (green) and LimEΔ (red) are higher than in the cytoplasm and accumulate throughout the hyaline zones. Average projection of 6 z-planes of the cells from bottom to top of the cells are shown. Time is indicated in seconds. Bar in the last frames, 10 μm. This figure corresponds to the movies shown in additional Movies S2-4

For line scans, the “Segmented Line” tool with 10 pixels width was used, and the data were processed in Excel, normalized to the maximum (Figs. 1 and 2).

## Results

### Chemotaxis of wild-type and coronin-null cells

To compare the chemotactic responses of *Dictyostelium* wild-type and coronin-null mutant cells, movement and reorientation of attached cells were recorded by application of gradients of the chemoattractant cAMP. The chemoattractant diffuses out of a micropipette and its position can be changed. To visualize the enrichment of filamentous actin and coronin, the wild-type cells expressed coronin-GFP and mRFP-LimEΔ (Fig. 1, and additional Movie S1).

LimE of *Dictyostelium discoideum* is a protein that is composed of a single LIM domain at its N-terminal region, followed by a central glycine-rich segment that comprises two repeat sequences, and a coiled-coil domain at its C-terminus. LimE is recruited to dynamic protrusions of the actin cytoskeleton, and is important for cytokinesis and cell motility [45]. Deletion of the coiled-coil tail domain leads to a reduction of cytoplasmic background when LimE is tagged with GFP or RFP at the C- or N-terminus. The fluorescent protein-tagged version of LimEΔ can be used as a marker for filamentous actin [56].

In wild-type cells, the coronin label localized to the inner border of the cortical actin layer at the leading edge as reported earlier [57], and LimEΔ accumulates at the front. To further analyze the distribution of coronin and LimEΔ, line scans of the fluorescence intensities corresponding to the single images are shown (Fig. 1). Using the same experimental chemotaxis setup with wild-type cells expressing coronin-GFP and mRFP-actin, a similar cortical localization of coronin is observed. However, in addition to the cell cortex, mRFP-actin also visualizes the cytoplasmic pool of actin (additional Fig. S1).

To investigate the chemotactic responses of the coronin-null cells, we used mutant cells that expressed GFP-tagged actin (green) and mRFP-LimEΔ (red) (Fig. 2A-C, and additional Movies S2-4). In contrast to wild type, coronin-null cells extend a broad convex hyaline zone enriched in actin-GFP and mRFP-LimEΔ when turning toward the direction of the gradient. This may indicate an increase in actin or an exclusion of the GFP probe from the endoplasmic reticulum (ER). In the line scans of the fluorescence intensities the increase of actin and LimEΔ in the whole area of the hyaline zones is seen (Fig. 2A-C). Figure 2A shows a cell that reacted toward the chemoattractant diffusing out from the pipette with a long, rounded hyaline zone. In Fig. 2B two cells respond with a round front turning the anterior hyaline zone into the direction of the gradient. Figure 2C shows as an additional detail during reorientation (70-s frame): the formation of several protrusions in various directions. Of these, the protrusion pointing to the new direction of the chemoattractant gradient expands and becomes the new front of the cell. It is obvious that when the front of the cell turns into the direction of the external gradient, the tail continues to move in the previous direction of the gradient of attractant.

A peculiarity of chemotaxing coronin-null cells is the almost complete absence of structured protrusions such as filopodia or lamellipodia from the large hyaline front region. Despite this absence, the cells efficiently orient and reorient in changing attractant gradients, with their rounded front region pointing into the direction of the current gradient (Fig. 2).

### The rounded protrusions in coronin-null cells are different from blebs

Figure 2 and additional Movies S2 to S4 show the chemotactic orientation and re-orientation of coronin-null cells that have extended hyaline front regions, which point toward the source of chemoattractant. With their rounded contours, these regions resemble blebs, but they are distinguished from them by the following criteria.

#### Actin filaments in the hyaline area of the coronin-null cells

**Fig. 3:**
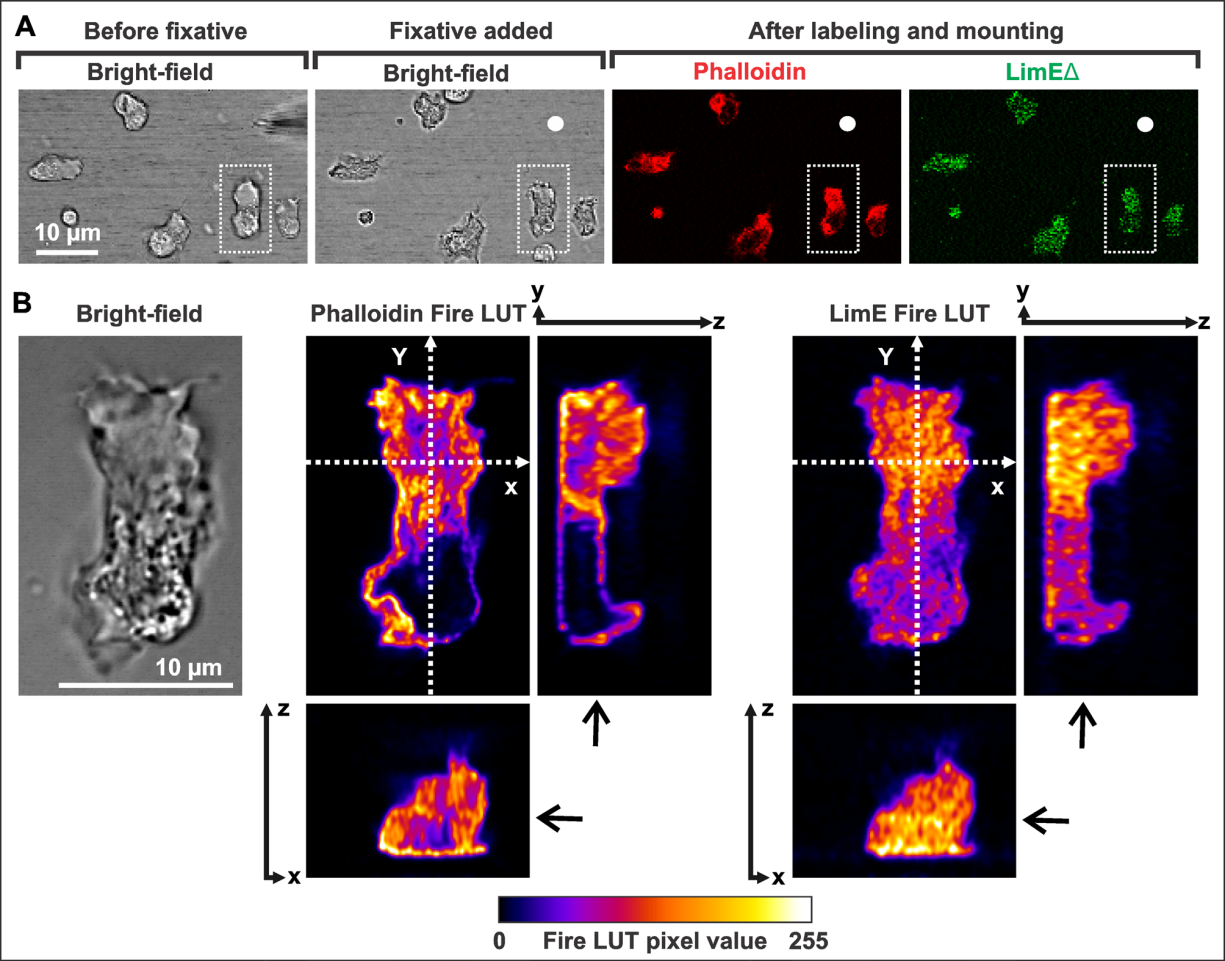
3D-distribution of phalloidin-label in coronin-null cells that were fixed during chemotaxis. The coronin-null cells used in the experiment expressed GFP-LimEΔ (green). (**A**) Overview of cells reacting to a gradient of cAMP that is diffusing out of the micropipette. The tip of the micropipette is marked with a white dot. From left to right, cells are shown before addition of fixative, after addition of fixative, and on the left, after labelling with phalloidin and mounting. The Alexa Fluor 568-phalloidin-label in red and the LimEΔ -label in green is shown. The cell marked with dotted lines is figured in B. (**B**) The phalloidin- and the LimEΔ -label of the marked cell in (**A**) is displayed in a fire LUT (Look Up Table). On the left, one plane of the bright-field is shown. In the middle, one confocal plane of the phalloidin-label, and on the right, one confocal z-plane of the LimEΔ-label is shown. On the bottom and right of these single planes xz- and yz-views through all z-planes of the original stack are presented and indicated by doted white arrows. Distance between the z-planes was 0.1 μm. The single z-planes out of the whole stack are indicated by black arrows. Time is indicated in seconds. Bars, 10 μm

To obtain direct evidence for the presence of filamentous actin in the hyaline zone of coronin-null cells, we stimulated mutant cells expressing GFP-LimEΔ with a micropipette, fixed them while they reorientated in the chemoattractant gradient, and labeled them with Alexa Fluor 568-phalloidin (Fig. 3A). Fixed coronin-null cells, labeled with fluorescent phalloidin showed filamentous actin within the lumen of the rounded protrusions as shown in the orthogonal views in Fig. 3B.

#### Independence from myosin II-activity

To test the dependence of the chemotactic response of coronin-null cells on the activity of myosin II, the cells were treated with 200 µM of (S)-3’-hydroxy-blebbistatin, which is a myosin II-activity blocker with excellent water solubility [53]. As a control, membrane blebbing was induced in wild-type AX2 cells by exposing them to 4 µM cAMP [39]. After testing different concentrations of (S)-3’-hydroxy-blebbistatin, the blebbing was efficiently inhibited by adding 200 µM (S)-3’-hydroxy-blebbistatin (Figure S2). Our results align with Zatulovskiy et al.‘s previous statement that coronin-null cells do not bleb [31] (see Table S1 in Ref. 31, position 24). After application of blebbistatin during chemotaxis experiments with coronin-null cells, the hyaline front zone of these cells did not shrink (Fig. 5).

### Non-requirement of substrate contacts for directed protrusions

**Fig. 4:**
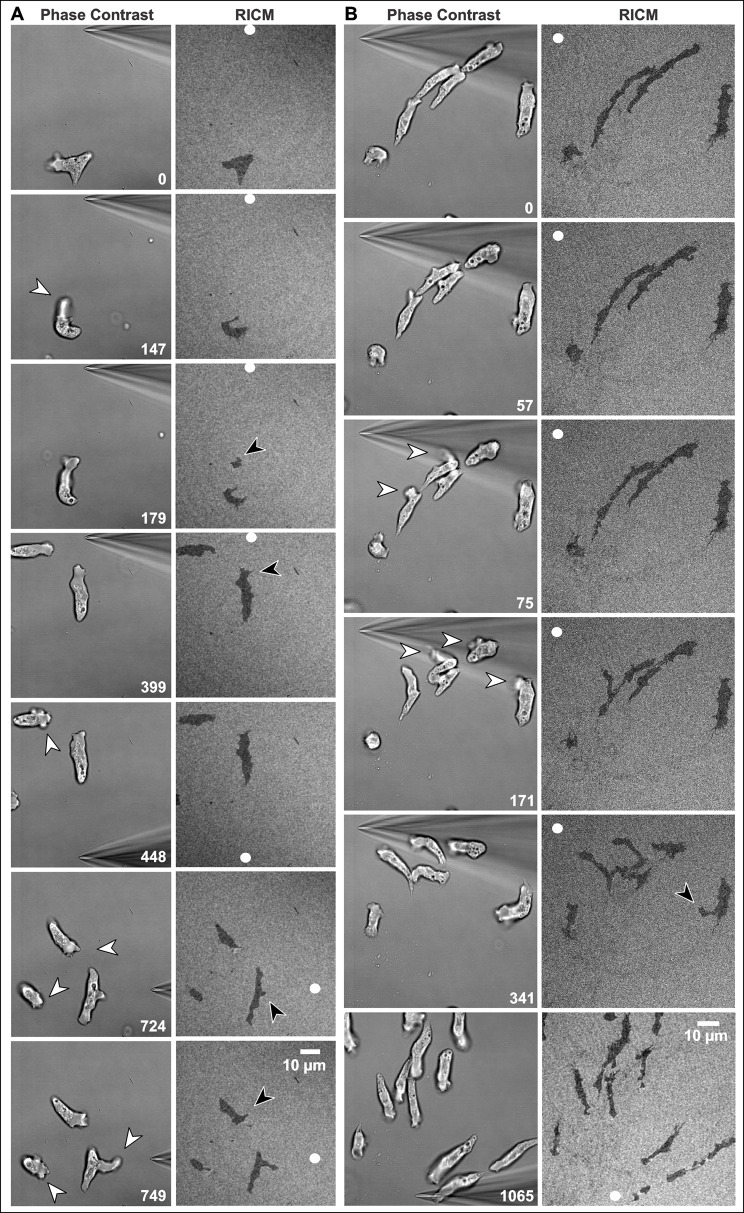
RICM of coronin-null cells in response to a gradient of chemoattractant. Cells were imaged by phase contrast and by total internal reflection microscopy (RICM). (**A**) and (**B**) In the two image-series, the left panels show phase contrast images of the cells stimulated with a micropipette filled with cAMP, and the right panels display the corresponding RICM images. The RICM images show only the parts of the cells that were attached to the substrate. The tip of the micropipette is marked with a white dot in these images. (**A**) In the 147-s frame, the hyaline zone above the substrate, in the 448-s frame many protrusions above the substrate after relocation of the pipette are seen. In the 724-s frame a small protrusion at the side of cell is formed in the direction of the pipette and in the 749-s frame this protrusion has been formed in a long hyaline zone to the chemoattractant in the pipette. (**B**) In the 57-s frame the cells reorientate toward the pipette at the left upper corner. At the beginning the old fronts are still attached to the substrate. In frames 75-s and 171-s the cells built new fronts that are above the substrate. Events where the front is not touching the substrate are marked with white arrow tips in the bright-field images. Events where protrusions are attached to the substrate are marked with black arrow tips in the RICM images. Time is indicated in seconds. Bar in the last frames, 10 μm. Figure 4A corresponds to the additional Movie S5

To study cell-to-substrate contact in relation to the chemotactic responses of coronin-null cells, we used reflection internal contrast microscopy (RICM). Confocal RICM images revealed that rounded protrusions formed and elongated in the direction of the chemoattractant without contacting the substrate surface (Fig. 4, additional Movie S5). This means that the mechanism that directs the chemotactic response does not need support from a solid surface.

### Further analysis of the hyaline front zones of coronin-null mutants in comparison to wild-type cells

**Fig. 5:**
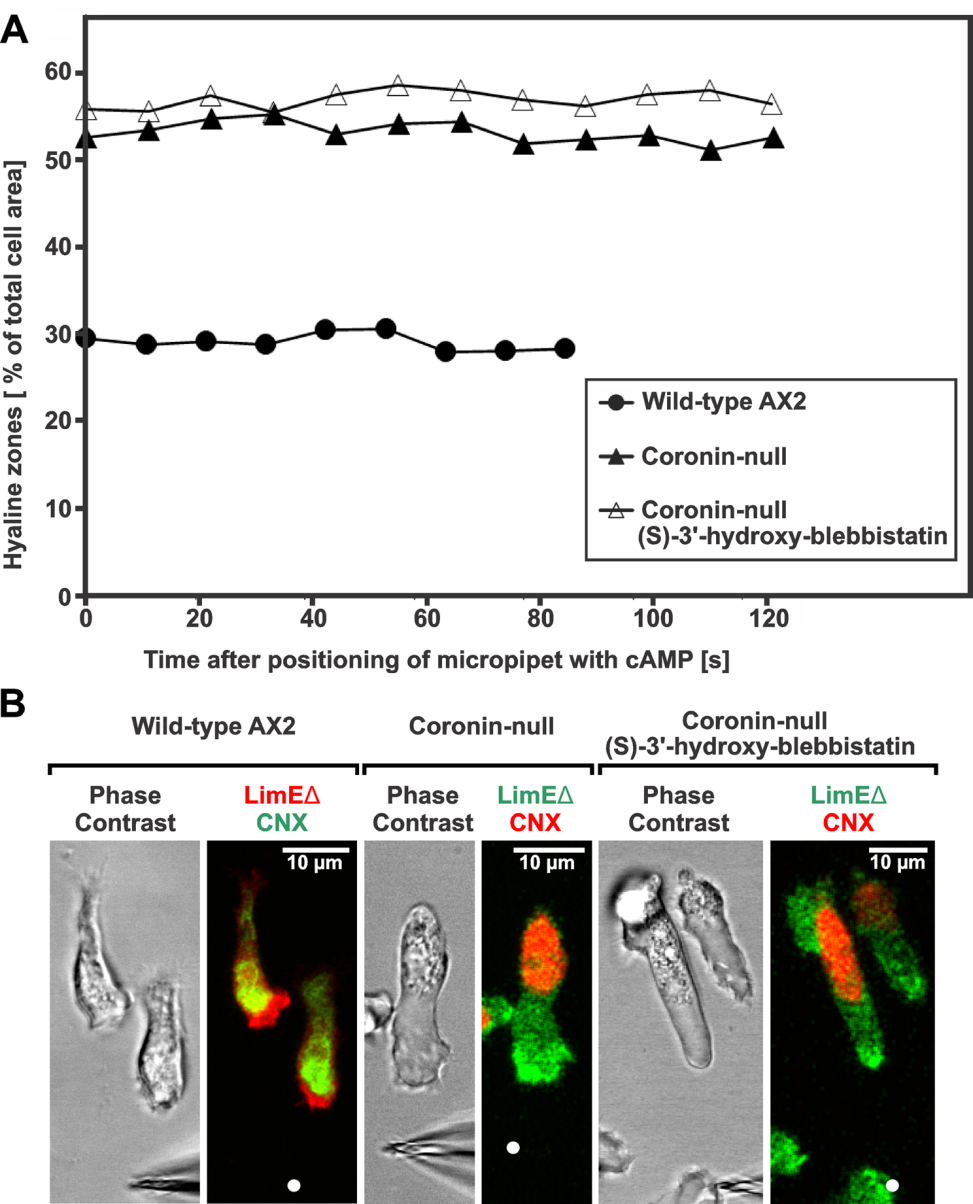
Size of the hyaline zone at the front region of chemotactically stimulated cells. (**A**) AX2 cells expressing GFP-calnexin (CNX) and mRFP-LimEΔ or coronin-null cells labelled with mCherry-calnexin and GFP-LimEΔ were used to determine the area that is free of the endoplasmic reticulum. Data points show the percentage of the area of the hyaline zone in respect to the whole cell area. The indicated time refers to the timepoint where the pipette with cAMP was positioned. Data points represent an average of 10 cells of AX2 wild type, 10 cells of coronin-null mutant, or 10 coronin-null mutant cells treated with 200 µM (S)-3’-hydroxy-blebbistatin. (**B**) From left to right: phase contrast image and merged fluorescence image of GFP-calnexin (CNX) and RFP-LimEΔ in AX2 wild-type cells, phase contrast and merged fluorescence image of calnexin-mCherry and GFP-LimEΔ in a coronin-null cell, and phase contrast and merged fluorescence images of CNX-mCherry and GFP-LimEΔ in a coronin-null cells treated with (S)-3’-hydroxy-blebbistatin. Cells react to a gradient of cAMP diffusing out of a micropipette tip. Bars, 10 μm. This figure is related to the movie shown in additional Movie S6

The localization of coronin to the inner border of the cortical actin layer at the leading edge (Fig. 1) is consistent with a role of coronin in actin depolymerization [23, 26, 57]. The large hyaline zone at the front region of coronin-null mutant cells would then be the consequence of a lack of this activity. We examined the front zones of AX2 wild-type and coronin-null mutant cells treated with the myosin II-inhibitor (S)-3’-hydroxy-blebbistatin in more detail (Fig. 5). To determine the size of this zone, we used a GFP- or mCherry-tagged version of calnexin (CNX). Calnexin is a membrane protein of the endoplasmic reticulum (ER), and the fluorescent marker for calnexin clearly visualizes the boundaries of the ER [58]; reciprocally it shows the expanded hyaline zone in coronin-null cells from which the ER is displaced. The areas of the whole cells and of the ER were determined, and the percentage of hyaline zones in respect to the total cell area were calculated using these areas. (Fig. 5A). The hyaline zones of wild-type AX2 cells represent approximately 29% of the total cell area. In contrast, the hyaline zones of untreated coronin-null cells comprise approximately 53% of the total cell area, and in (S)-3’-hydroxy-blebbistatin inhibitor-treated coronin-null cells, the hyaline zones comprise approximately 57% of the total cell area (Fig. 5A).

In Fig. 5B we show examples of the front zones of starved AX2 wild-type cells, of the hyaline zones of starved coronin-null cells and of coronin-null cells treated with (S)-3’-hydroxy-blebbistatin which react to a cAMP gradient coming out of a micropipette. Wild-type cells were labelled for filamentous actin using mRFP-LimEΔ and for calnexin using GFP-CNX, and coronin-null cells using GFP-LimEΔ and mCherry-CNX. Aggregation-competent cells were elongated and oriented towards the micropipette tip. Compared to AX2 wild-type cells, the actin-rich hyaline zone in relation to the ER is much larger in the coronin-null cell and after treatment with blebbistatin these zones are not diminished and even slightly larger than in untreated coronin-null cells (Fig. 5A, B).Starved, aggregation-competent coronin-null cells treated with 200 µM (S)-3’-hydroxy-blebbistatin for 1 h could still orient in a gradient of cAMP released from a micropipette. We measured the velocity of the reaction of the front zone and it was markedly slowed down in comparison to untreated coronin-null cells (Table 2, and additional Movie S6).

**Table 2:** Average velocity of directional front movement of coronin-null mutant cells stimulated with cAMP through a micropipette, without and with (S)-3’-hydroxy-blebbistatin treatment

Cell stain	Velocity in µm/min	Standard error
Coronin-null	22.80	± 0.05
Coronin-null (S)-3’-hydroxy-blebbistatin	13.80	± 0.03

## Discussion

*Dictyostelium discoideum* cells lacking coronin A differ from wild-type cells in that they have a large, hyaline area at their front end. As we demonstrate, this area is rich in filamentous actin and lacks organelles, particularly the endoplasmic reticulum. Despite this unusual morphology of the front region and the impaired actin turnover [15, 25, 26, 28], coronin-null cells manage to orientate in a gradient of chemoattractant by extension of rounded protrusions from the hyaline area.

A characteristic of the chemotactic motility of coronin-null cells is the dispensability of differentiated cortex structures such as lamellipodia or filopodia. The mutant cells can move by rounded protrusions formed by the directed expansion of their hyaline area (Fig. 2, additional Movies S2-4). During reorientation of a cell, the formation of several such protrusions is observed. These protrusions compete with each other, and the one pointing toward the source of the attractant becomes the new propagating front (Fig. 2C). Our results demonstrate that these protrusions differ from blebs in several respects. Chemotactic movement of *Dictyostelium* wild-type cells is considered to be mainly driven by polymerization of F-actin, however, bleb formation was described as an alternative mechanism to generate force under specific conditions [36, 39, 59]. Blebs are formed in wild-type cells by the rupture of membrane-cortex connections under the contractile activity of myosin II [59].

We also addressed the question how motility is powered in coronin-null cells and tested the effect of 200 µM (S)-3’-hydroxy-blebbistatin, a concentration that completely blocked the myosin II-dependent blebbing in wild-type cells. Coronin-null cells treated with this inhibitor showed that the hyaline zones are not diminished and slightly larger than in untreated cells suggesting an additional major contribution provided by actin polymerization. However, treated cells can still orient in the cAMP gradient (Fig. 5, and additional Movie S6), but the velocity of chemotactically stimulated coronin-null cells treated with the myosin II-inhibitor blebbistatin in comparison to untreated cells was reduced by roughly one-third. It is known that the chemotaxis efficiency of myosin II-null mutant cells is impaired due to reduced cell polarity and minimized translocation speed as a consequence of the inability to retract the uropod [60]. This could also explain the reduction of the velocity and the slightly larger hyaline zones of coronin-null cells.

Furthermore, by staining with phalloidin we found the round protrusions in coronin-null cells to be populated by filamentous actin. The enrichment of the actin-GFP label in the hyaline zones of coronin-null cells supports this finding. In contrast to this, in blebbing cells actin can only be found in the cell cortex and as scars at the border of the blebs. The level of actin in the blebs is the same as in the cytoplasm [35, 39]. As an example, the level of actin in the temporally and new made protrusion in Fig. 2C (Frames 70-s and 177-s) are higher than in the cytoplasm. The chemotactic migration of coronin-null cells is therefore different from a bleb-driven mode described for *Dictyostelium* [31].

As shown by RICM, the cells orient with their large hyaline zones in response to the gradient of the attractant and elongate the rounded fronts in the direction of the gradient without attachment to the glass surface and being in a confined environment.

In summary, *Dictyostelium* cells lacking coronin can move efficiently in a chemotactic gradient by a mechanism that differs both from actin-driven lamellipodia formation in wild-type cells and bleb formation which is found under certain conditions. Coronin-deficient cells thus provide an example of how the absence of certain actin-regulating proteins can lead to further variations and alternative forms of chemotactic movement.

## Conclusions

**Fig. 6:**
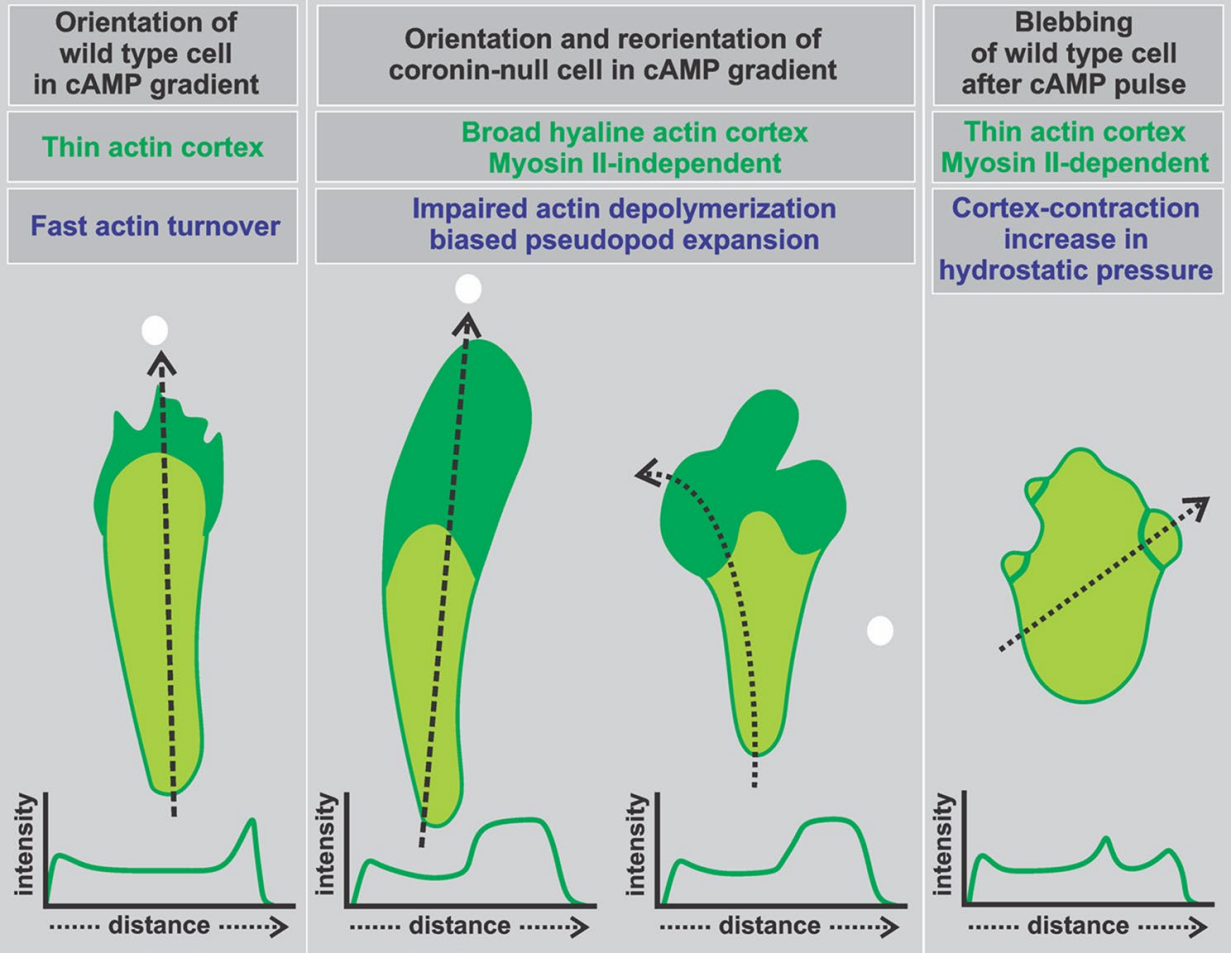
Scheme depicting the mode of pseudopod extension of wild-type and coronin-null cells reacting in a chemotactic gradient in comparison to bleb formation in wild-type cells stimulated by a cAMP pulse. Left: A wild-type cell moving in a cAMP gradient. Filamentous actin accumulates and is continuously turned over at the front. Pseudopods and filopodia are extended toward the gradient. Middle: A coronin-null cell reacts with its large, rounded hyaline zone to a cAMP gradient and during reorientation makes small protrusions before reacting with one of these to the new gradient. Due to impaired actin filament turnover and reduced depolymerization, filamentous actin is enriched in the broad hyaline front zones. Inhibition of myosin II does not reduce the hyaline zones. Right: A wild-type cell stimulated by a cAMP pulse. Cortex-contraction is followed by the increase in hydrostatic pressure and causes bleb-formation (adapted by Langridge and Kay et al., 2006). This process is myosin II-dependent. The highest concentration of the gradient is marked with a white dot. Schematic scans of corresponding actin distribution through the cells are shown at the bottom

*Dictyostelium discoideum* cells lacking coronin A were shown previously to be impaired in actin-polymerization. These mutant cells respond to a chemoattractant by formation of rounded protrusions from their large hyaline front regions. We re-investigated this unusual mode of migration and show that the hyaline region is devoid of organelles and filled with filamentous actin. We provide evidence that the mode of directed migration in chemotactic gradients of coronin A-mutant cells is different by several criteria from blebbing, which is an alternative to pseudopodium-based migration under specific conditions and the preferred mode of movement of *Dictyostelium* cells or granulocytes in confined 3D environments. These findings are summarized in a conceptual model in Fig. 6.

## Supplementary Information

Below is the link to the electronic supplementary material.

Supplementary Material 1: Additional Movie S1. Chemotaxis of aggregation-competent AX2 wild-type cells

Supplementary Material 2: Additional Movie S2. Chemotactic reorientation of coronin-null cells that expressed actin-GFP

Supplementary Material 3: Additional Movie S3. Chemotaxing coronin-null cells

Supplementary Material 4: Additional Movie S4. Turning of chemotaxing coronin-null cells

Supplementary Material 5: Additional Movie S5. RICM of coronin-null cells responding to gradients of chemoattractant diffusing out of a micropipette tip

Supplementary Material 6: Additional Movie S6. Chemotaxis of coronin-null cells expressing LimEΔ-GFP (green) and calnexin-mCherry (red) treated with 200 µM (S)-3'-hydroxy-blebbistatin for 1 hour

Supplementary Material 7: Additional Information: Additional Figures S1and S2 with legends and additional Movie legends. PDF-format

## Data Availability

Data and Materials are available from the corresponding author (ME) upon request.

## Declarations

### Author’s information

Günther Gerisch passed away in June 2025 after completing and approving the main part manuscript.
