# Supplementary material for: Chemotactic motion of coronin-null cells with impaired actin turnover
Source: BMC Mol Cell Biol. 2026 Mar 26;27:20. doi: 10.1186/s12860-026-00585-9 (PMC13064307; doi:10.1186/s12860-026-00585-9)
Supplement: Supplementary file 7 — Supplementary Material 7: Additional Information: Additional Figures S1and S2 with legends and additional Movie legends. PDF-format [file 12860_2026_585_MOESM7_ESM.pdf]

Additional Information

Additional Figures and Legends

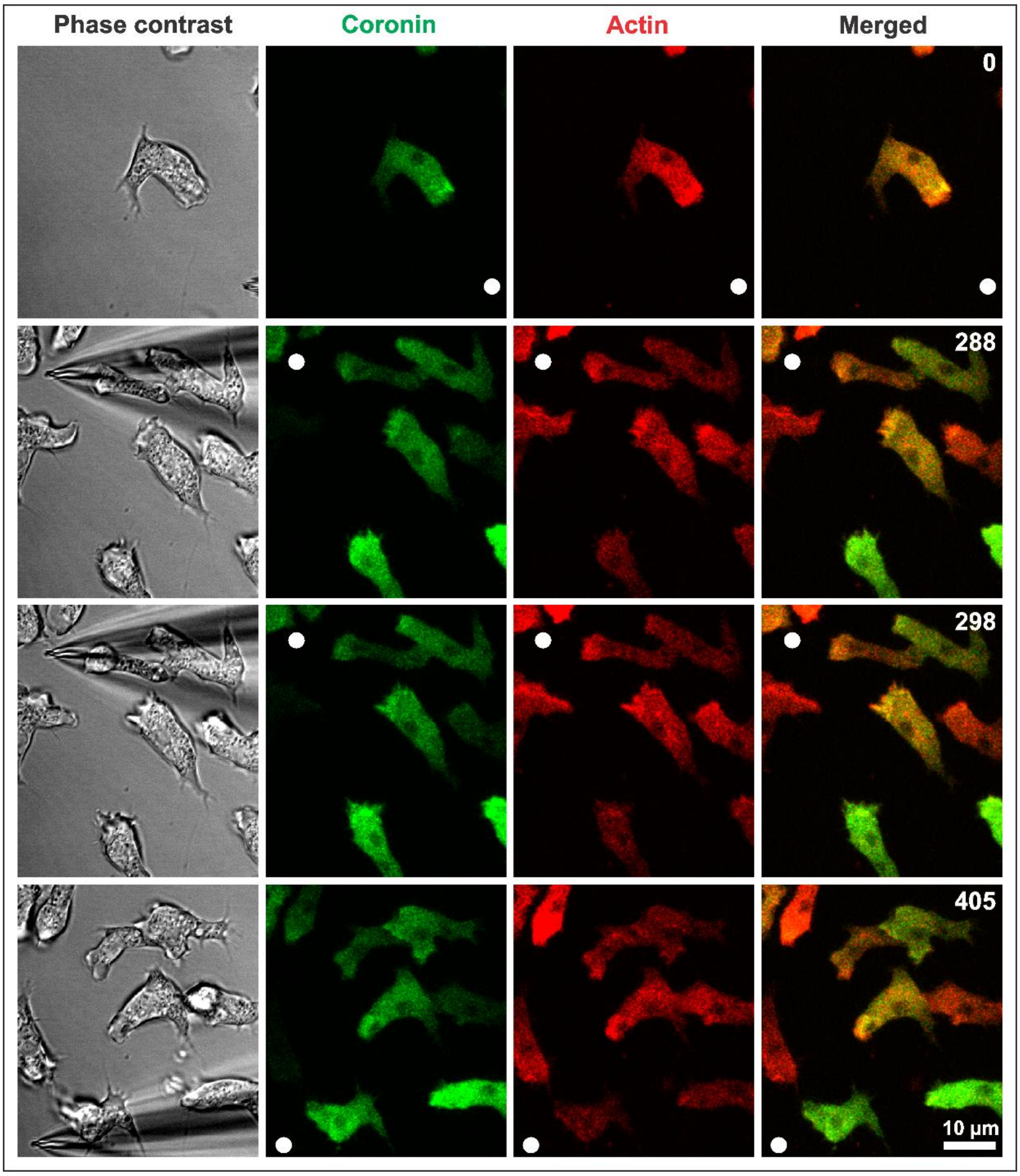

Figure S1. **Chemotaxis of aggregation-competent AX2 wild-type cells expressing coronin-GFP (green) and mRFP-actin (red).** The cells react to a cAMP-gradient that is built around the tip of the micropipette. After repositioning of the pipette, the cells reorient according to the changing gradient. The tip of the micropipette is marked with a white dot. Actin is accumulating at the front, while coronin can be seen slightly offset behind actin. From the bottom to the top, average projections of six z-planes of the cells are shown. Time is indicated in seconds. Bar, 10 µm.

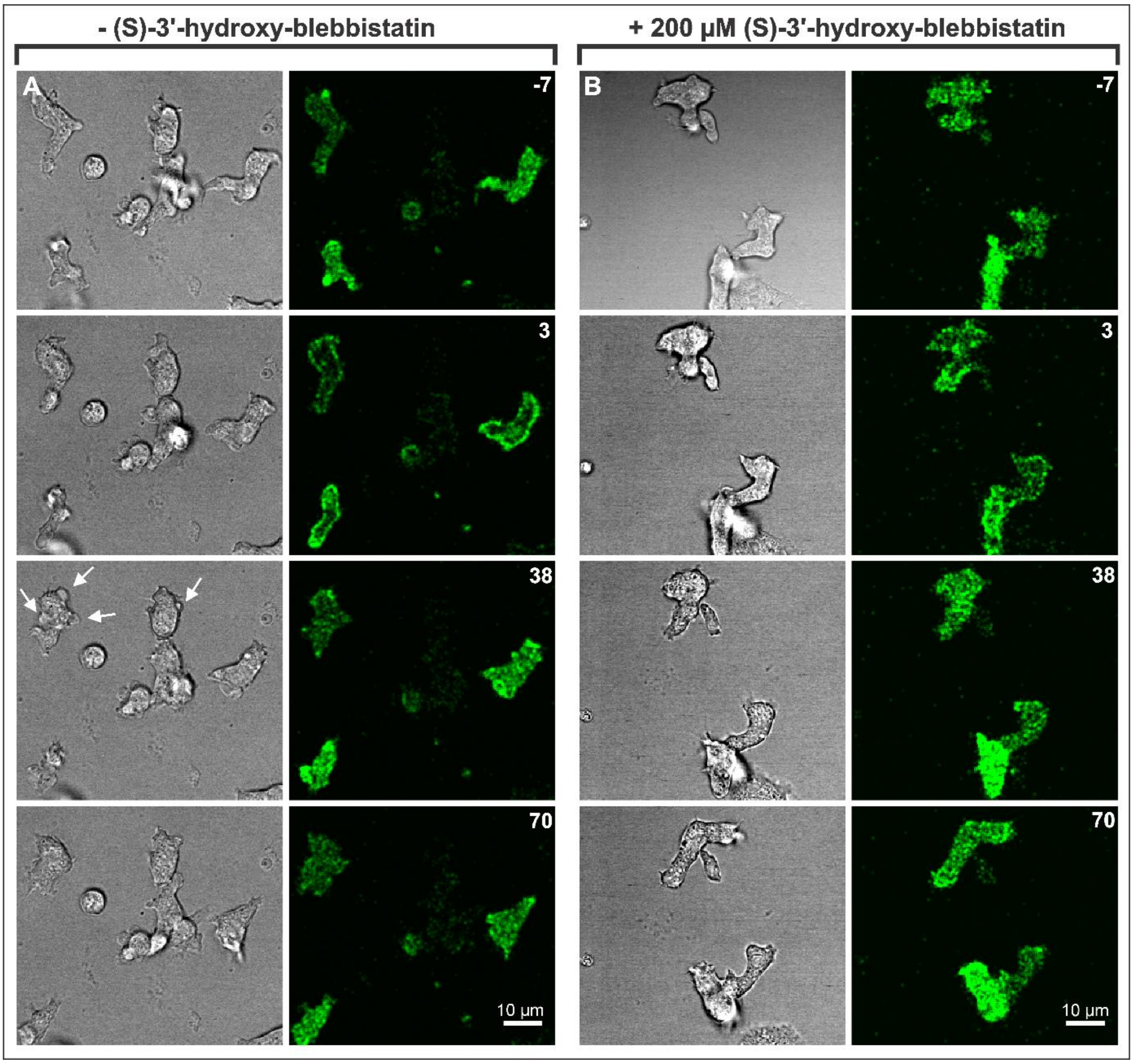

Figure S2. **Chemoattractant stimulation of AX2 wild-type cells. Blebbing and suppression of blebbing in the absence (-) and presence (+) of (S)-3'-hydroxy-blebbistatin.** Cells expressing GFP-LimEΔ were starved for 6 hours. (A) Cells without (S)-3'-hydroxy-blebbistatin treatment. 38 s after a pulse with 4 µM cAMP, blebbing can be observed (white arrows in phase-contrast channel). (B) In cells treated with 200 µM (S)-3'-hydroxy-blebbistatin, no blebbing occurred. The change in F-actin distribution can be observed in the GFP-channel of (A) and (B) at about 3 s in both treated and untreated cells. Time is indicated in seconds. Bars, 10 µm.

Additional Movie Legends

Movie S1. **Chemotaxis of aggregation-competent AX2 wild-type cells.** The cells expressed coronin-GFP (green) and mRFP-LimEΔ (red). The cells reacted to a cAMP-gradient that is built around the tip of a micropipette. The tip of the micropipette is marked with a white dot in the fluorescence images. After repositioning of the pipette, the cells reorient. Coronin is accumulating slightly behind the LimEΔ-labeled actin at the front. This additional movie is related to Figure 1.

Movie S2. **Chemotactic reorientation of coronin-null cells that expressed actin-GFP (green).** One cell responded to the chemoattractant gradient with a large hyaline zone that is clearly enriched in actin-GFP. The tip of the micropipette is marked with a white dot. This additional movie is related to Figure 2A.

Movie S3. **Chemotaxing coronin-null cells.** The cells expressed actin-GFP (green). Two cells respond with a round front turning the anterior hyaline zone into the direction of the gradient. The tip of the micropipette is marked with a white dot in these images. This additional movie is related to Figure 2B.

Movie S4. **Turning of chemotaxing coronin-null cells.** The cells expressed actin-GFP (green). Upon changing the position of the micropipette, the tail continued to move in the previous direction of the gradient, while the front region initially made protrusions into multiple directions, and finally one expanded toward the new gradient. The tip of the micropipette is marked with a white dot. This additional movie is related to Figure 2C.

Movie S5. **RICM of coronin-null cells responding to gradients of chemoattractant.** The cells were labeled with CNX-mCherry as a marker for the endoplasmic reticulum. The left panels display bright-field only. In the middle, the bright-field image (blue) is overlaid with a CNX-label (red). On the right side, the RICM images show only the parts of the cells that are attached to the substrate. This additional movie is related to Figure 4A.

Movie S6. **Chemotaxis of coronin-null cells expressing LimEΔ-GFP (green) and CNX-mCherry (red) treated with 200 μM (S)-3'-hydroxy-blebbistatin for 1 hour.** The cells show efficient reaction to the gradient of cAMP diffusing from the pipette tip, and reorient upon relocation of the pipette. The large hyaline zones have not decreased in size during reorientation. This movie corresponds to Figure 5A and B.
